# Dosimetric accuracy of low dose rate volumetric modulated arc therapy delivery in an Elekta Versa HD linear accelerator for pulsed low dose rate treatment of recurrent high‐grade glioma using Monaco planning system: Low dose rate VMAT‐based PLDR in Elekta Linac

**DOI:** 10.1002/acm2.70253

**Published:** 2025-09-03

**Authors:** Surendran Jagadeesan, S. P. Vijaya Chamundeeswari

**Affiliations:** ^1^ Department of Physics, School of Advanced Sciences Vellore Institute of Technology Vellore Tamilnadu India; ^2^ Department of Radiation Oncology Burjeel Cancer Institute Burjeel Medical City Abu Dhabi UAE

**Keywords:** high‐grade glioma, Low dose rate delivery, pulsed low dose rate (PLDR), Re‐irradiation, VMAT

## Abstract

**Background and purpose:**

Reducing the dose rate enhances efficacy in radiation therapy by allowing increased repair of sub‐lethal damage. Pulsed low‐dose radiation therapy (PLDR) is an innovative approach that is safe and effective for the reirradiation of recurrent gliomas and radioresistant tumors. In this study, the accuracy of the low dose rate volumetric modulated arc therapy (VMAT) delivery is tested in an Elekta Versa HD linear accelerator (linac) for delivering PLDR.

**Material and methods:**

A retrospective study was conducted on ten patients previously treated for high‐grade glioma. The Monaco planning system was used to create two or three arc VMAT plans with a minimum monitor unit (MU) exceeding 570. The Mosaiq record and verification system (R&V) was used to select dose rates between 20 and 25 MU/min for delivering VMAT beams, achieving a beam‐on time of 30 min and an effective dose rate of 6.7 cGy/min. Measurements from IBA MatriXX resolution were used for gamma analysis. Beam characteristics at low dose rates for 8 gantry angles were investigated using the measurements from the dolphin detector.

**Results:**

All the deviations of flatness and symmetry were within 1.0% in all gantry angles. MU linearity was within 1% for the lowest dose rate of 20 MU/min. The output deviations were within 1% for all dose rates. The average dose rate achieved was 6.7 ± 0.1 cGy/min. The maximum dose rate achieved in the planning target volume (PTV) was 7.28 (6.86 to 7.28) cGy/min. The gamma pass rate was above 97%, with the analysis criterion 3%3 mm, 2%2 mm, and 2%1 mm for VMAT delivered at a low dose rate.

**Conclusion:**

Low‐dose rate VMAT delivery is feasible with higher accuracy in the delivered dose on the Elekta Versa HD linac. PLDR can be implemented successfully on Elekta linacs using the Monaco planning system.

## 1 INTRODUCTION

Reducing the dose rate improves efficacy in radiation treatment as it enables a greater degree of repair of sublethal damage.^1^, ^2^, ^3^ The overall beam‐on‐time, as opposed to the average linear accelerator (linac) dose rate or the instantaneous dose rate (IDR), determines the dose‐rate effect in external beam radiotherapy utilizing the linac.^4^ Relative to three‐dimensional conformal radiotherapy (3DCRT), the treatment duration is prolonged in intensity‐modulated radiotherapy (IMRT). The introduction of volumetric modulated arc therapy (VMAT) has shortened the beam‐on time much less than the IMRT. A study on assessing dose rate distributions on VMAT shows that the mean dose rate hardly exceeds 100 cGy/min in the planning target volume (PTV) for conventionally fractionated head and neck and cranial irradiations.^5^ Dose rate frequency distribution quantified by time resolved Monte Carlo simulations in another study reveals that in VMAT, up to 75% of the PTV can have its dose delivered with dose rates < 100 cGy/min.^6^

Treatment options for unresectable recurrent gliomas are constrained, mainly as most recurrences occur locally within a previously irradiated region.^7^ Re‐irradiation can improve palliative outcomes; however, there are concerns about normal tissue toxicity. Pulsed low‐dose‐rate radiotherapy (PLDR) is a new technique proposed for the reirradiation of recurrent gliomas.^8^, ^9^, ^10^, ^11^ This technique improves tumor kill via dose rate effects, resulting in low‐dose hyper‐radiosensitivity (low‐dose HRS) of the tumor when the radiation dose is below a transition dose.^8^, ^10^, ^12^ Studies have indicated that the transition dose is equal to or below 30–50 cGy in tumor cells and less than 20 cGy in normal tissues.^8^ This low‐dose HRS phenomenon increases the therapeutic index by improving cell death in proliferating tumor cells while reducing its effects in non‐proliferating normal tissues. In traditional PLDR, the daily conventional prescribed dose of 200 cGy is divided into 20 cGy subfractions (pulses) and delivered at certain time intervals within a single session. The time interval between pulses is based on the clinical practicability to achieve an effective low dose rate of ∼6.7 cGy/min to maximize the normal tissue repair process.^8^, ^22^ It is important to mention that the term “pulse” is used to describe a fraction of the daily dose or a fraction of the total monitor unit (MU) of the treatment beam and not the electric pulse produced by the accelerator's pulse forming network (PFN).

Several studies have reported the clinical efficacy of PLDR for recurrent cancers, unresectable sarcomas, malignant central nervous system (CNS) tumors, and patients in palliative conditions.^14^, ^15^, ^16^, ^17^, ^18^, ^19^ Results show that the tumor control probability (TCP) could be significantly enhanced for glioma cell lines with PLDR.^8^ Reirradiation with PLDR is more effective and well‐tolerated for the thorax, abdomen, and pelvis.^24^ A recent prospective phase II clinical study administered PLDR for recurrent nasopharyngeal carcinoma and reported that a combination of systemic therapy and PLDR can provide survival benefits with less toxicity.^20^

In the first introduced PLDR method, the 200 cGy fractional dose is divided into ten radiation pulses of 20 cGy, delivered over 30 min with 3‐min intervals between each pulse to achieve a low dose rate (6.7 cGy/min) effect.^8^ Dividing the total MU to create pulses is relatively more straightforward in 3DCRT techniques. However, the plan quality will be suboptimal. A highly conformal treatment plan with the lowest possible dose to the surrounding critical structures is desired for reirradiation. Several studies reported implementing PLDR using IMRT or VMAT techniques.^13^, ^21^, ^22^, ^23^, ^24^, ^25^, ^26^ In an IMRT‐based study using Elekta linac, five subfractions (40 cGy per subfraction) were used to deliver 200 cGy, and the treatment duration was 50 min, resulting in an effective dose rate of 4 cGy/min.^13^ The RapidArc treatment technique with two arcs was used in a study that achieved an effective dose rate of 6.7 cGy/min. Each arc delivered a mean dose of 20 cGy and a maximum dose of < 40 cGy.^21^ The linacs (Elekta, Siemens, and Varian) used in these studies were operated at MU rate 50–100 MU/min.^13^, ^21^, ^22^, ^23^, ^24^

The Varian TrueBeam linac can run as low as 5 MU/min. A study demonstrated the delivery accuracy of Step and Shoot IMRT in the TrueBeam accelerator with a low dose rate in the order of 5, 10, 15, and 20 MU/min for PLDR delivery.^27^ The Elekta Versa HD linac can deliver a dose rate ranging from 12 MU/min to 600 MU/min using the continuous variable dose rate (CVDR) mode. A recent study investigated the PLDR delivery efficiency in Elekta Versa HD linac using low dose rate mode. The study achieved the PLDR effect through continuous low‐dose‐rate VMAT delivery without beam interruptions at a dose rate of 25 MU/min, resulting in an effective dose rate of 6.8 ± 0.1 cGy/min.^28^

Despite several publications on its benefits, PLDR techniques are still not widely implemented due to limitations in planning systems that include the selection of dose rate and MU limits in plan optimization. Due to complex multileaf collimator (MLC) segments and gantry movements, delivering PLDR using advanced techniques was challenging. Dividing the total beam MU by 10 to create dose pulses in IMRT or VMAT plans introduces many low MU segments, which may affect the delivery accuracy and sometimes not deliverable. Maintaining the sub‐fractional gaps is another drawback of this approach.^28^ Elekta's Monaco (Elekta AB, Sweden) planning system combines the gold‐standard Monte Carlo algorithm and is designed to meet the challenges of modern radiation oncology.^29^ The segment shape optimization (SSO) in VMAT planning is the key advantage of the Monaco planning system, which allows to shape dose distributions accurately without hardcoding MU limits and dose rate in the plan. To date, there have been no published reports regarding the PLDR utilizing VMAT planning created with the Monaco planning system. This retrospective study investigates the accuracy of the low dose rate VMAT using Elekta Versa HD linac and the Monaco planning system for delivering PLDR.

## 2 MATERIALS AND METHODS

### 2.1 Beam characterization

Output, MU linearity, flatness, and symmetry were measured at low dose rates of 20, 30, 40, 50 MU/min and a maximum dose rate 600 MU/min to investigate the beam characteristics. Machine output were measured for field size 10 × 10 cm^2^ at eight gantry angles as follows 0°, 45°, 90°, 135°, 180°, 225°, 270°, 315°. MU linearity measurements were performed for 10 × 10 cm^2^ field using an in air cylindrical farmer chamber IBA FC65G with acrylic buildup cap for MUs 2, 3, 4, 5, 10, 20, 25, 50, 75, and 100 to generate a linearity curve and calculate the slope. Flatness and symmetry were measured for field size 30 × 30 cm^2^ at eight gantry angles. Output, flatness and symmetry measurements were performed with a dolphin detector mounted to the linac head. Measurement analysis was performed using the MyQA software (IBA Dosimetry, Germany). The dolphin detector measured counts for 10 × 10 cm^2^ field size, and 100 MU was calibrated against the machine output measured using the cylindrical farmer chamber in a water phantom at the isocenter. Flatness and symmetry were analyzed using the formula as per IEC 60976, and the values for all gantry angles were compared with values measured at gantry angle 0°.

### 2.2 Treatment delivery in Elekta linac

Mosaiq v 2.81 (Elekta, Stockholm, Sweden) handles treatment delivery, record, and verification (R&V) for Elekta linacs. The selected beam for delivery in Mosaiq is sent to the linac treatment control station (TCS) via iCOM field transfer. When the treatment beam is transferred, the TCS automatically calculates the dose rate (MU/min) for each segment of the field based on the prescribed movement of all geometric parameters in relation to the prescribed dose to keep the beam delivery time as short as possible. If the calculated dose rate is lower than the selected one, the TCS uses the calculated dose rate for delivery. If the actual dose rate deliverable in the machine is less than 75% of the dose rate chosen, it can cause the beam to terminate. It should be noted that in the Elekta linac, there will be inter segment breaks during VMAT delivery where only movement occurs with no dose however the TCS keeps the beam delivery time shorter by selecting higher MU rates for segments. As the TCS automatically selects the most applicable dose rate value for delivery, it is not necessary to prescribe a dose rate in the planning system when the treatment plan is created for Elekta linacs.

**FIGURE 1:**
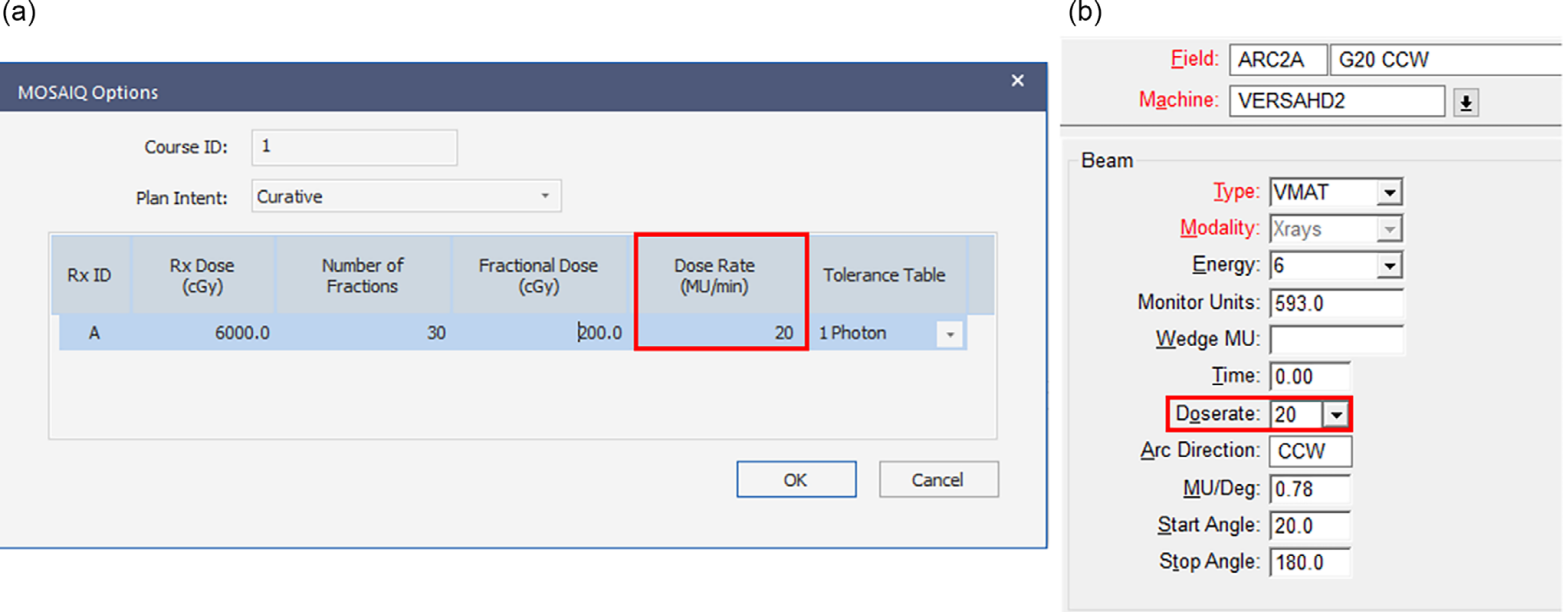
(a) Option for dose rate selection for delivery during plan export in Monaco planning system and (b) Option for dose rate selection under field properties in Mosaiq.

Monaco has the option of selecting dose rate for IMRT and VMAT delivery during the plan export to Mosaiq. When desired the intended dose rate for delivery can be entered (Figure 1a) in the “Dose Rate (MU/min)” cell. Alternatively, the dose rate can be added to the field after importing the plan in to Mosaiq under the field parameters (Figure 1b). For a standard treatment, the maximum dose rate of the machine is selected so that the delivery takes place over the wide range of nominal dose rates. When a dose rate below the nominal value (600 MU/min) is selected, it is set as a maximum dose rate for the delivery to the TCS. Therefore, dose rates below the set value are used for the treatment delivery.

In Elekta linacs, dose rate calibration and gun current optimization are performed to achieve the nominal maximum dose rate for the machine during commissioning and routine service and maintenance. A similar approach was followed to find the machine's lowest possible deliverable dose rate. We attempted to achieve a dose rate down to 15 MU/min. However, the minimum deliverable dose rate we could achieve on our two‐beam matched machine was 20 MU/min. Dose rates below this value are used during beam startup, sometimes for fewer segments in a VMAT delivery. However, the entire VMAT treatment could not be delivered below this dose rate.

### 2.3 Treatment planning and delivery for PLDR

Ten patients previously treated for high‐grade glioma using the VMAT technique were selected for this retrospective study. The treatment plans were created to achieve the normal tissue constraints for conventional fractionations with a prescription dose of 6000 cGy delivered in 30 fractions. All these plans were made using a 6 MV photon beam. Based on the laterality of the tumor location, either partial or full arc VMAT beams are used. The start angle for arcs was 180 degrees, and the arc size used was 200 to 240 degrees for partial arcs and 360 degrees for full arcs. The minimum segment width used was 0.5 cm, and fluence smoothing was Low. Beamlet width used for optimization: 0.3 cm. A calculation uncertainty of 0.5 % and a 3 mm dose grid were used for dose calculation. Segment shape optimization is enabled during plan optimization, as recommended by Elekta, to account for the maximum possible distance the gantry can travel between control points based on the machine's available dose rate.

Our preliminary testing showed that for the Elekta Versa HD linac, the average beam loading time was 0.6 min, which includes the time for setting the linac MLC leaves, gantry, collimator for delivery, and beam start‐up time. This means that when two single arc VMAT beams are selected for delivery, 0.6 min are added to the total delivery time. Equation (1) below shows the simple expression of the calculation of the PLDR effective dose rate.

(1) $$\mathrm{PLDR}\;\mathrm{Effective}\;\mathrm{Dose}\;\mathrm{rate}=\frac{\mathrm{Prescribed}\;\mathrm{Dose}}{\mathrm{Delivery}\;\mathrm{time}}\;$$

### 2.4 The delivery time is defined as

(2) $$\begin{array}{cc} & \mathrm{Delivery}\;\mathrm{time}\\ & \;=\mathrm{Beam}\;\mathrm{loading}\;\mathrm{and}\;\mathrm{startup}\;\mathrm{time}+\mathrm{Beam}\;\mathrm{on}\;\mathrm{time} \end{array}$$

where the beam‐on time is the duration that the beam remains on.

With the prescription dose of 200 cGy, the total delivery time from the beam start should be 30 min to achieve an average dose rate of 6.7 cGy/min.

The beam‐on time shall be calculated according to Equation (3)

(3) $$\mathrm{Beam}\;\mathrm{on}\;\mathrm{time}=\lfloor \frac{\mathrm{Total}\;\mathrm{MU}}{\mathrm{MU}\;\mathrm{Rate}}\rfloor \;$$

Our goal was to achieve a delivery time of 29 to 30 min, to attain the effective dose rate for PLDR. When substituting the values for a typical VMAT plan delivering ≥ 570 MU and a selected MU rate of 20 MU/min in equation (3), the beam‐on time is approximately:

$$\frac{570\;\mathrm{MU}}{20\;\mathrm{MU}/\min}=28.5\;\min$$

Adding the beam loading and startup time (∼0.6 min for two arcs) gives a total delivery time of approximately 29.1 min, yielding an effective dose rate of:

$$\frac{200\;\mathrm{cGy}}{29.1\;\min}=6.87\;\mathrm{cGy}/\min$$

This calculation ensures that the effective dose rate does not exceed ∼ 6.9 cGy/min, resulting in a real‐time dose in the PTV that is less than the transition dose for low‐dose HRS. It is also essential that the maximum dose rate at any point within the PTV is below the transition dose, ∼ 40 cGy per pulse. This corresponds to a real‐time maximum dose rate of 13.3 cGy/min for a beam‐on duration of 30 min. When the maximum dose in the target is 210 cGy and the beam‐on time is 30 min, the maximum point dose rate is ≤ 7 cGy/min. Therefore, a maximum point dose limit in the target has been established during planning, which should not exceed 105% (210 cGy per fraction) of the prescribed dose.

For seven out of ten patients selected in this study, a new plan was created as the original plans had MUs lower than 570. Depending on the planning target volume (PTV), geometry, and plan complexity, two‐arc or three‐arc VMAT fields were used. For PTVs smaller than 150 cm^3^, we used four arcs in the replan to achieve the delivery constraints for PLDR. The plan optimization harmonized equal and uniform dose contribution to the PTV volume from each VMAT arc field used. In the re‐planning process, our objective was to increase the total plan MU above 570 and achieve highly conformal dose distributions while adhering to organ‐at‐risk (OAR) constraints. Several parameters were adjusted during the re‐planning, including changing the arc increment, segment shape parameters, and controlling the extent of the 50% isodose line, which eventually helped to achieve a highly conformal plan with an average MU above 570. Multicriterial optimization (MCO) is used in stage one optimization to achieve the lowest extent of the 50% isodose.

Following the treatment plan, MU rates were computed using equation (3) and this value was set during the plan export to Mosaiq (Figure 1a) for delivery. The service graphing tool in the TCS used to record the treatment log files at the sampling rate of 250 ms. This treatment log files are analyzed using in‐house developed software to assess the delivery time, dose rate, MLC, and gantry angle positions. The variation and reproducibility of the gantry and the central MLC leave positions during low‐dose‐rate delivery are compared with the machine's maximum dose rate delivery. The maximum dose deposited by each arc in the PTV was extracted from the planning system to calculate the maximum dose rate per arc. The effective dose rate and the maximum dose rate per arc was calculated using equation (1).

### 2.5 Verification measurements and analysis

IBA MatriXX resolution with mini phantom is used for patient‐specific quality assurance. The MatriXX resolution consists of a matrix of 1521 detectors 0.016 cm^3^ vented plane‐parallel ionization chambers spaced 6.5 mm center‐to‐center, giving a total area of 25.3 cm × 25.3 cm. The effective measurement plane (EMP) for MatriXX is 3.5 mm below its surface. The multicube mini phantom R comprises RW3 and has a 38 × 32.1 × 14.4 cm dimension. An inclinometer is attached to the gantry to monitor the gantry angle, which is used for angular response correction. The matrix device was cross‐calibrated for field size 10 × 10 cm^2^ to generate a user correction factor for the measured dose plane.

All the measurements for quality assurance were performed in a single setup to avoid any random variations in measurement. The coronal dose plane was measured for the selected low dose rate to compare with the TPS‐calculated dose plane and the measured dose plane for the beam delivered at the maximum dose rate. Gamma analysis was performed using the criterion 3%3 mm, 2%2 mm, 2%1 mm, and 1%1 mm.

## 3 RESULT

**FIGURE 2:**
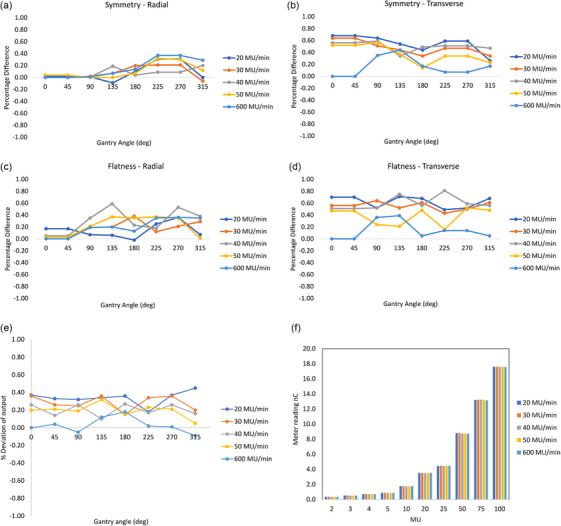
(a) and (b) symmetry variations in radial and transverse direction for eight gantry angles and five dose rates. (c) and (d) flatness variations in radial and transverse direction for eight gantry angles and five dose rates. (e) output deviations for eight gantry angles and five dose rates. (f) MU linearity for different MUs and five dose rates.

The overall variations of flatness and symmetry were within 1% for all the measurements with varying dose rates and gantry angles. Figure 2a‒2d shows the flatness and symmetry deviations along the radial and transverse direction for eight gantry angles and five dose rates. The symmetry variations were slightly higher in the transverse direction (Figure 2b) than in the radial direction (Figure 2a), attributed to the servo system, which controls the beam in the radial direction for the linear accelerator. The flatness and symmetry variations were within the specifications of AAPM TG 142. The variations of output were within 0.5% for all the measurements (Figure 2e). A linear regression model was applied to the MU linearity curve (Figure 2f) for all dose rates, which yielded a slope value of 0.1768 ± 0.0006 with *p = 0.0001* and R^2 ^= 1.

Table 1 provides the summary of ten patients PTV volume, number of ARC, total MU, MU per arc, MU rate selected for PLDR delivery, total delivery time, and the average and maximum dose rate in the PTV. The PTV volume ranged from 94.97 to 599.14 cm^3^. The average dose rate achieved was 6.7 ± 0.1 cGy/min. The average plan MU was 611 ± 29. The average delivery time was 30 ± 0.5 mins. The maximum dose rate achieved in the PTV was 7.28 cGy/min (range: 6.86 to 7.28 cGy/min). The maximum point dose to PTV and OARs achieved in the plans are summarized in Table 2.

**TABLE 1:** – PTV Volume, ARC geometry, MU, Beam dose rate, delivery time, average and maximum dose rate statistics.

Patient	PTV Volume (cm^3^)	Number of ARC	Total MU	MU per ARC				Beam delivery dose rate MU/min	Total delivery time (min)	PTV Average dose rate (cGy/min)	PTV Maximum point dose rate (cGy/min)
				ARC1	ARC2	ARC3	ARC4				
1	342.76	3	629.55	218.33	223.04	188.18	–	21	30.1	6.6	6.98
2	94.97	4	633.00	155.7	66.21	214.58	196.61	22	29.7	6.7	7.27
3	237.72	3	589.00	184.15	198.34	206.71	–	20	29.9	6.7	7.17
4	154.64	4	592.00	129.65	137.04	132.51	192.8	20	29.8	6.7	7.04
5	269.34	2	601.10	296.13	304.97	–	–	20	30.2	6.6	6.93
6	126.18	4	572.02	135.34	177.41	112.32	146.95	20	29.5	6.8	7.28
7	442.74	3	644.29	210.66	224.48	209.15	–	21	31.0	6.4	6.86
8	254.28	3	605.00	199.92	228.26	176.82	–	20	30.6	6.5	6.88
9	346.80	2	582.67	263.24	319.43	–	–	20	29.3	6.8	7.22
10	599.14	2	659.00	325.93	333.07	–	–	22	30.1	6.6	7.05

**TABLE 2:** – DVH statistics.

Patient	Maximum dose (cGy)										Mean Dose (cGy)	
	PTV	Brainstem	Optic chiasm	Spinal cord	Rt. Lens	Lt. Lens	Rt. Optic nerve	Lt. Optic nerve	Rt. Eye	Lt. Eye	Rt. Cochlea	Lt. Cochlea
1	6277.8	5491.5	2819.6	138.9	360.8	425.1	1421.1	1361.6	924.8	796.7	382.5	396.4
2	6274.1	4153.9	1974.4	112.5	363.6	406.7	1139	645.5	716.4	476.8	101.7	149
3	6332.6	5298.5	4679	121.3	473.9	531.9	3555.8	1951.7	1840.1	853.5	2789.2	1178.3
4	6248.2	5617	5311.1	378.4	418	424.8	3005.4	3182.9	1141.5	1142.2	1982.4	2714.4
5	6246.6	5146	5310.2	131.2	460.2	596.3	2970.8	5068.3	1637.5	2356.5	4352.6	778.2
6	6248.6	5534.9	5449.8	185.8	521.3	537.9	5493.5	4602.3	1628.1	1669.9	4406.4	1519.8
7	6309.8	5594.4	5149.2	176.8	479.2	532.2	2546.7	5108.6	1077.4	2302.8	1403.5	4465.6
8	6247.9	5212.5	5175.6	167.2	512.1	491.7	4145.5	3678.7	1548.1	1367.2	264.1	185.8
9	6313.4	4867.3	2952.5	136.1	480.7	527.8	1399.5	2824.5	805.6	1856.2	920.9	3200.6
10	6338.8	5389.2	5493.3	125.7	413.3	512.3	5306.5	5293.1	4348	4000.2	361.7	365.9

**FIGURE 3:**
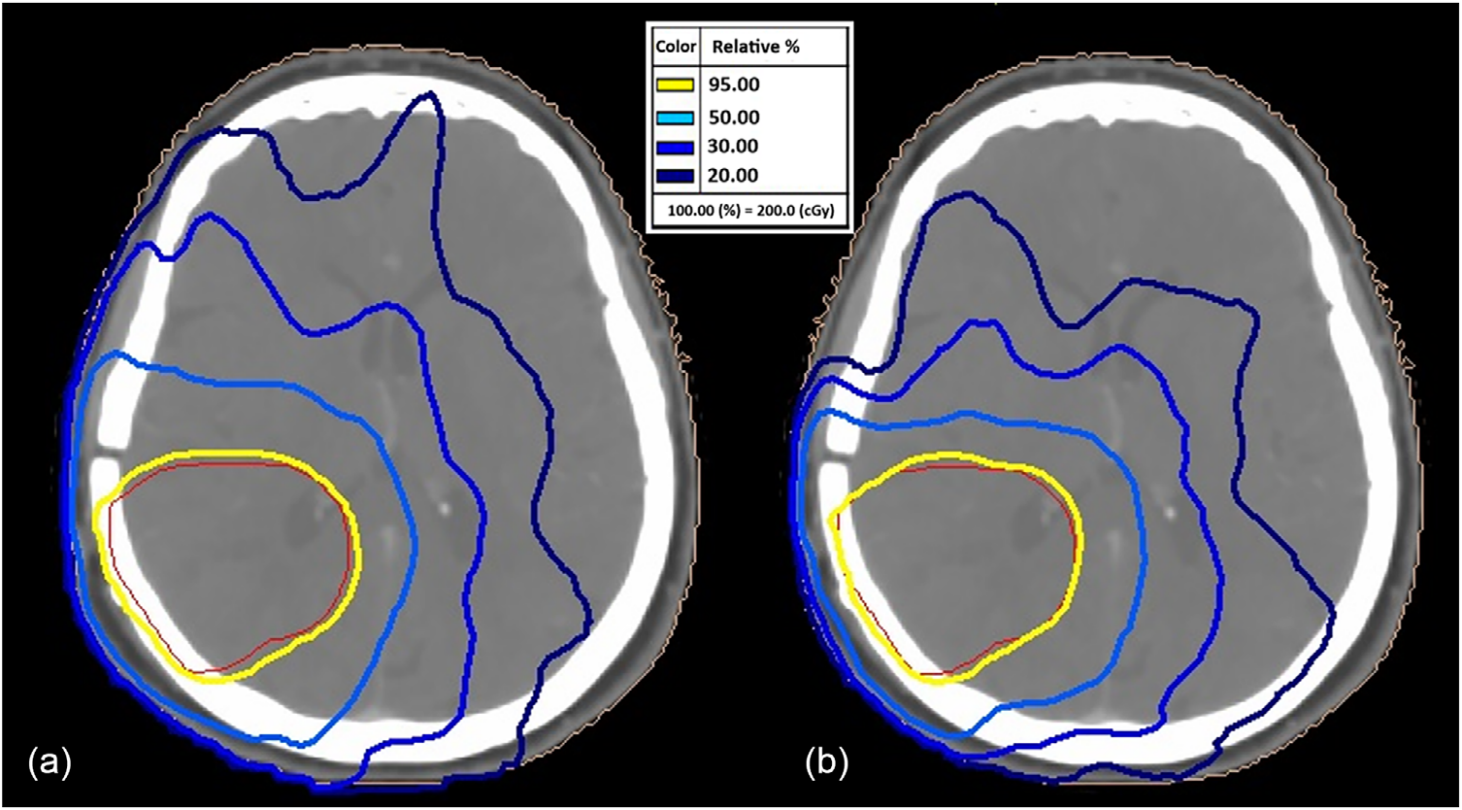
95%, 50%, 30%, and 20% isodose line distributions for a two‐arc initial VMAT plan with 381 MU (a) and a four‐arc VMAT replan 633 MU (b) for a PTV of volume 94.97 cm^3^.

Figure 3 illustrates a comparison of isodose line distributions for (a) Initial two‐arc plan delivering 381 MU and a replanned four‐arc VMAT delivering 633 MU for a planning target volume (PTV) of 94.97 cm^3^. The replan with 633 MU has a comparatively lesser volume of 50%–20% isodose than the plan with 381 MU, which is clinically advantageous in a re‐irradiation setting.

**TABLE 3:** – Patient‐Specific Quality Assurance Results.

Patients	TPS calculated vs. measured (600 MU/min)				TPS calculated vs. Measured (20 MU/min)			
Gamma criteria →	3%/3 mm	2%/2 mm	2%/1 mm	1%/1 mm	3%/3 mm	2%/2 mm	2%/1 mm	1%/1 mm
1	99.8	99.4	97.3	79.8	100	99.4	98.9	80.1
2	100	99.6	99.6	88.6	100	99.7	99.7	87.8
3	100	100	99.8	90.3	100	100	99.9	89.5
4	100	99.4	97.9	81.3	100	99.6	98.5	82.5
5	100	99.8	99.5	90.7	100	99.7	99.6	90.5
6	100	99.8	98.4	86.4	100	99.8	99.2	87.2
7	100	99.5	98.6	84.5	100	99.7	99.7	84.8
8	100	99.8	99.1	89.5	100	99.8	99.6	88.7
9	100	99.2	98.2	86.2	100	99.4	99.4	87.4
10	100	99.6	99	87.8	100	99.8	99.5	86.9

**FIGURE 4:**
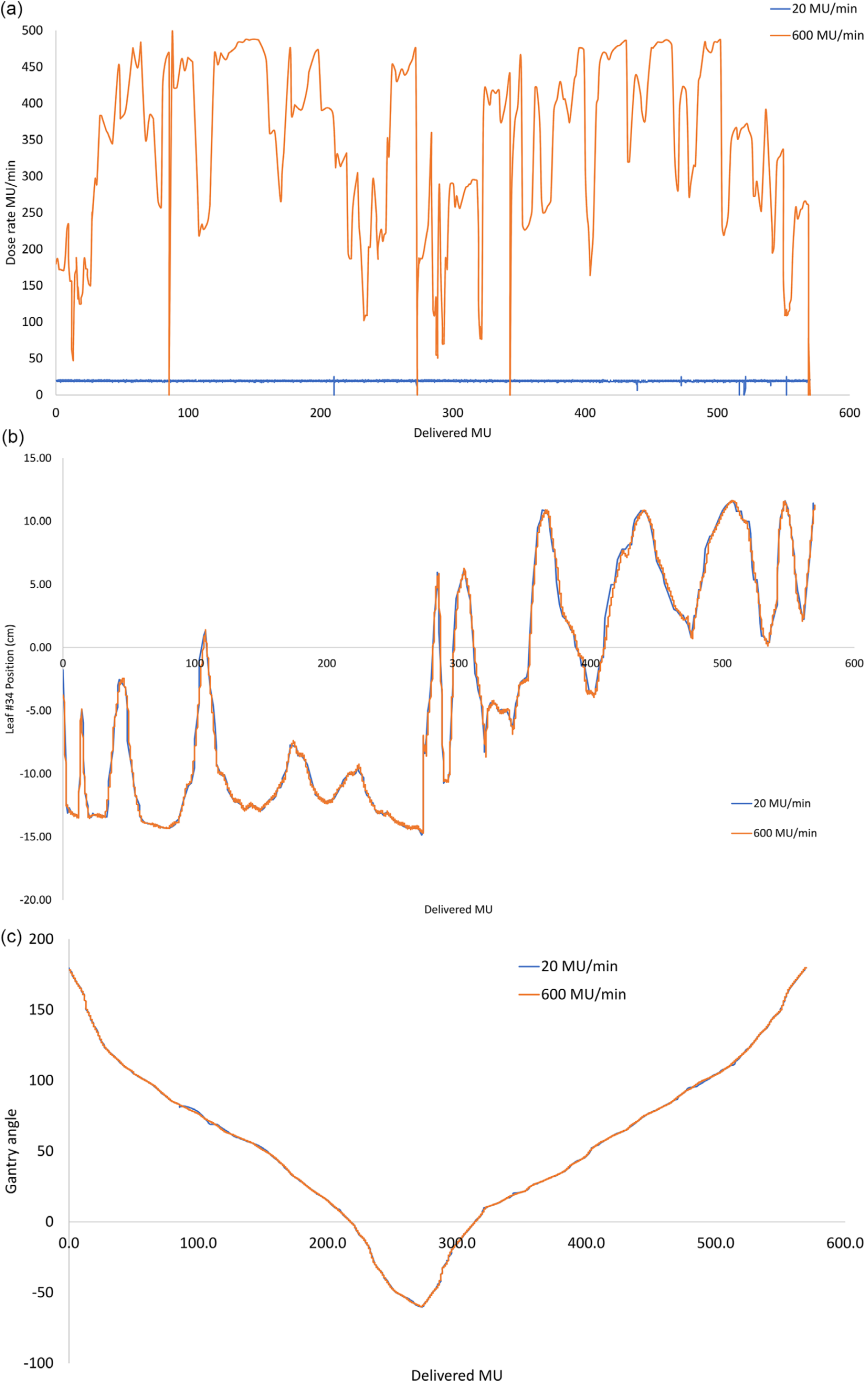
(a) The dose rate variations (y‐axis) during VMAT delivery for 600 MU/min and 20 MU/min against delivered MU (x‐axis). (b) The central MLC leaf #34 positions (y‐axis) during delivery for 600 MU/min and 20 MU/min against delivered MU (x‐axis). (c) The gantry angle positions (y‐axis) during delivery for 600 MU/min and 20 MU/min against delivered MU (x‐axis).

**FIGURE 5:**
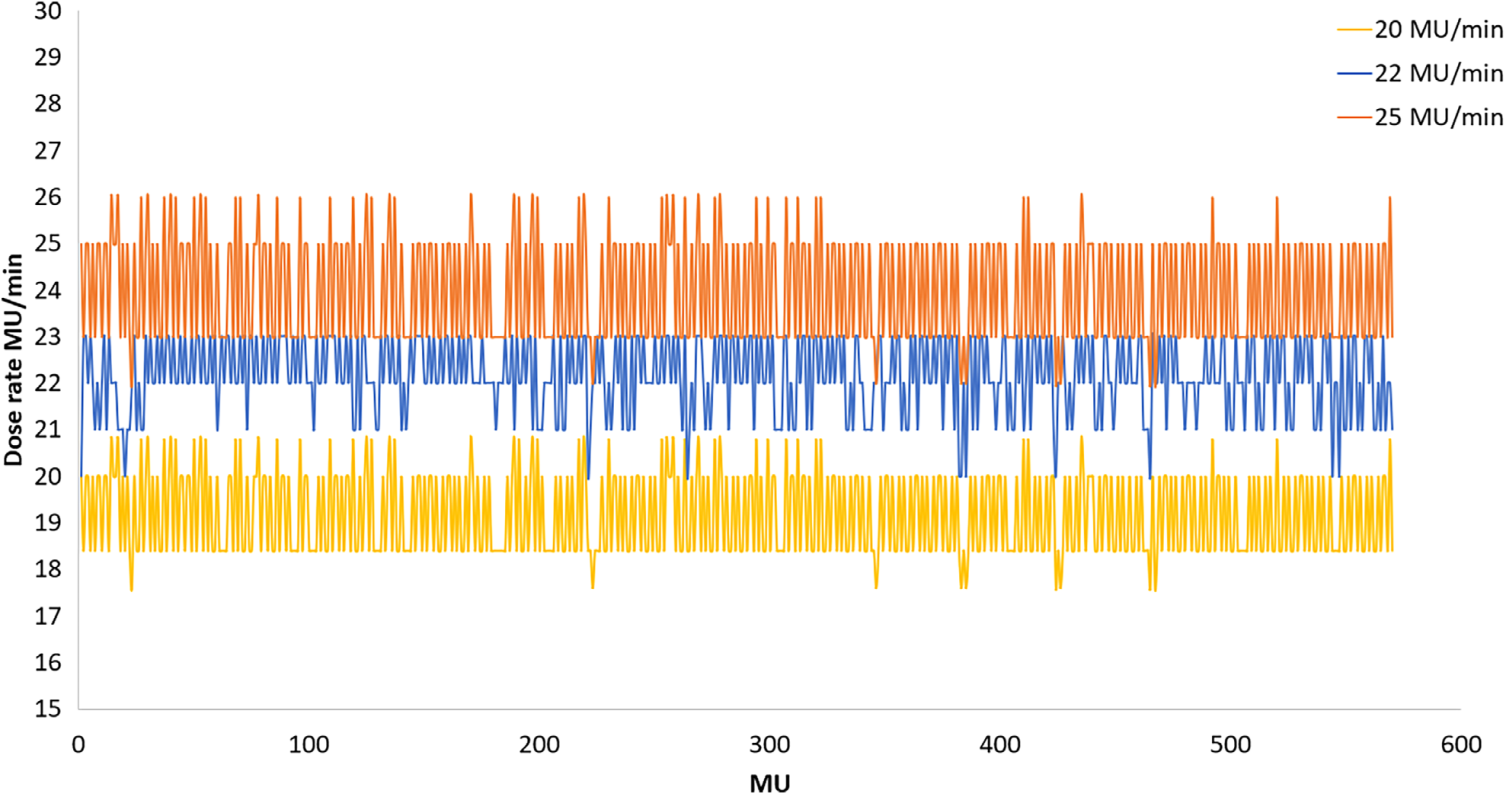
Dose rate variations during delivery when the maximum dose rate was limited to 20, 22, and 25 MU/min (y‐axis) against delivered MU (x‐axis).

Figure 4 plots the dose rate variations, central MLC #34 position, and gantry angle changes as a function of the MU delivered for the same VMAT plan when delivered with maximum dose rate set to 600 MU/min and 20 MU/min respectively. The dose rate variations during delivery with dose rates 20 MU/min and 600 MU/min are shown in Figure 4a. The central MLC leaf and gantry angle positions for the two dose rates are shown in Figure 4b, c. These characteristics were similar for all the patients in this study. The average dose rate was 285.8 ± 139.07 MU/min when the maximum dose rate during delivery was 600 MU/min. When the maximum dose rate was limited to 20, 22, and 25 MU/min, the average beam delivery dose rate achieved was 19.9 ± 0.4, 22.1 ± 0.8, and 24.0 ± 1.1 MU/min, respectively. Figure 5 shows the dose rate variation during delivery when the maximum dose rate was limited to 20, 22, and 25 MU/min for the same VMAT plan.

**FIGURE 6:**
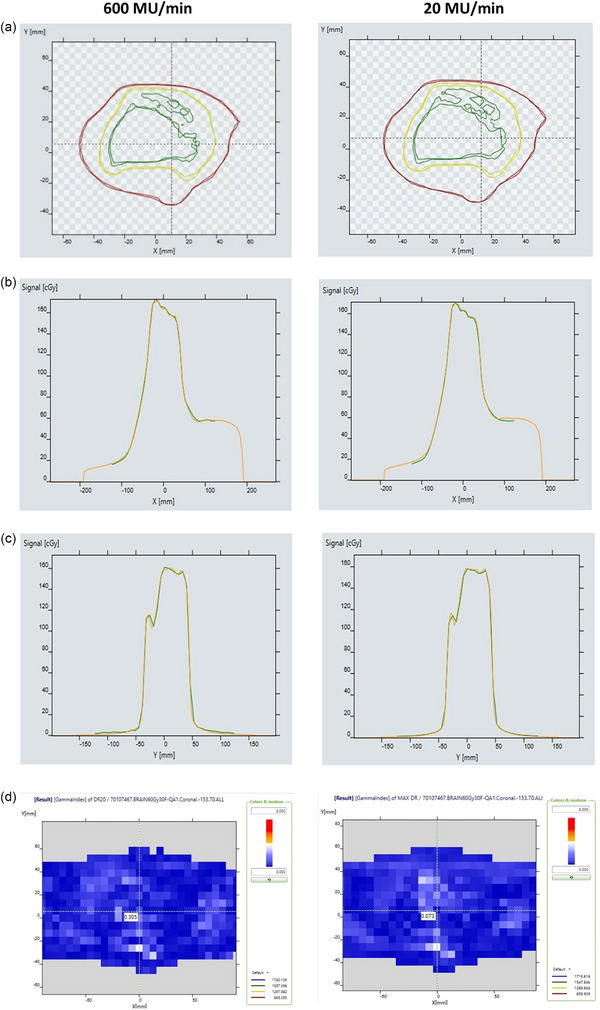
(a) Isodose line comparison between measured and TPS calculated coronal dose plane, (b) transverse dose profile comparison, (c) radial dose profile comparison, (d) gamma distribution map for the dose rate 600 MU/min and 20 MU/min respectively.

Gamma pass rates (GPR) were more than 99% for all the patients with criteria 3%/3 mm and 2%2 mm when the TPS dose plane is compared with the measured dose plane for the maximum dose rate of 600 and 20 MU/min (Table 3). When the gamma criteria changed to 2%1 mm, the GPR was 98.7 ± 0.8 % and 99.4 ± 0.4 % for the dose rates 600 and 20 MU/min, respectively. The GPR for the criteria 1%1 mm was 86.5 ± 3.7 % and 86.8 ± 3.4 % for the dose rates 600 and 20 MU/min, respectively. An example of gamma comparison results between measured and calculated coronal dose plane is shown in Figure 6 for the two dose rates.

## 4 DISCUSSION

In this study we have investigated the feasibility of low dose rate VMAT delivery and accuracy in an Elekta Versa HD linac for implementing PLDR. Our study is the first to use VMAT plans created on the Monaco planning system for pulsed low dose rate delivery. Our study uses a continuous low‐dose‐rate VMAT delivery strategy to achieve the same biological effect as traditional PLDR, resulting in an effective dose rate of approximately 6.7 cGy/min without the need for explicit beam interruptions or sub‐fractionation. PLDR delivery with subfractions poses some practice difficulties and is not a convenient method.^28^ Our method avoids the practical challenges of beam interruptions, which is easily practicable and more effective. It improves delivery efficiency while maintaining the radiobiological intent of PLDR.

A feasibility study for delivering PLDR utilized Elekta Axesse linac with beam modulator, and VMAT planning was achieved using Pinancle^3^ 9.0 SmartArc algorithm (Philips Radiation Oncology Systems, Fitchburg, WI). The VMAT plan MU for 200 cGy is divided by 10 in the record and verification system and delivered with a 3‐min beam gap to achieve the average dose rate of 6.7 cGy/min.^23^ In that study, the minimum MU per degree and the leaf speed parameters were modified during the planning to make the VMAT beam deliverable after reducing the beam MU by a factor of 1/10. This necessitates having two machine profiles with different MLC parameters in the planning system to create VMAT plans for standard dose rate and PLDR delivery. Furthermore, constraining the leaf speed parameters must be handled with caution as it may have higher modulation and complexity in the plan which may lead to lower pass rates.^23^, ^39^

The first study reporting a continuous low‐dose rate PLDR VMAT delivery on Elekta Versa HD linac used the Eclipse TPS (Varian Medical Systems, Palo Alto, CA) to create VMAT plans.^28^ Eclipse allows the selection of dose rates and minimum and maximum MU limits during plan optimization. Therefore, the dose rate was set at 25 MU/min, and the minimum and maximum MU limits were set at 570 and 630 during VMAT planning in the study. The actual achieved dose rate during treatment delivery was 22 ± 0.09 MU/min, and the effective dose rate achieved was 6.88 ± 0.1 cGy/min. Although the method proposed in this study is effective, Eclipse TPS is not commonly utilized for planning in facilities equipped with Elekta linac. Studies reported that implementation of the PLDR technique is limited due to the lack of a planning system that supports PLDR planning.^22^, ^28^

Elekta supplies the Monaco planning system, a dedicated solution for planning with Elekta machines, which is widely available. Monaco provides superior VMAT plans with a variable dose rate when SSO is enabled during plan optimization.^29^ Although Monaco does not support setting MU limits, the required minimum MU for the plan shall be achieved using the other optimization tools in the system. Monaco does not support dose rate selection in VMAT planning when SSO is enabled. However, in Elekta linacs, the dose selection for delivery can be managed in the TCS; hence, it is not required to be considered during the planning.^30^ As long as we can achieve the minimum plan MU above 570, we can select the dose rate for delivery based on the effective dose rate needed for the PTV. The effective dose rate achieved in our study was 6.7 ± 0.1 cGy/min, and the maximum dose rate was 7.07 ± 0.2 cGy/min. Our maximum point dose rate was much lower than the previously published values.^23^, ^28^ In our study, the initial plan MUs for small PTVs (< 150 cm^3^) were around 380–450 with two ARCs. In such cases, the plan was re‐created using additional arcs (up to four) to meet the MU requirement for PLDR delivery, while maintaining target coverage and OAR constraints. This was intricate in selecting the arc field and the optimization parameters. However, during our initial planning studies, we have established the required optimization strategies for achieving plan with MU above 570. Therefore, the planning time to achieve a VMAT plan that meets the PLDR delivery constraints for delivery was comparable to standard plans.

The TCS in Elekta linacs is handled by integrity software, which used a binned dose rate ranging from 37 to 600 MU/min in previous versions. From Integrity Software version 4.0.0, the CVDR option is available.^31^ This option enables a wide range of dose rate selections in small steps, allowing smoother and faster delivery without compromising the dosimetric accuracy.^31^, ^32^ Some studies reported that the CVDR offers a higher average beam dose rate and dosimetric advantage but, at the same time, may affect the positioning accuracy of gantry and MLC due to increased speed.^32^, ^33^, ^34^, ^35^ On the other hand, low dose rate delivery drives the gantry and MLC at a very low speed, which may also affect the positioning accuracy.^36^ TG 218 recommends using tighter criteria such as 2%1 mm or 1%1 mm as it is susceptible to identifying subtle regional errors.^37^ However, there is no recommended acceptable level of GPR for the criteria 1%1 mm because the dosimetric error and statistical fluctuations are dominant.^38^ The GPR in our study for the criteria 2%1 mm shows that the delivery accuracy is superior in low‐dose rate VMAT delivery. The pass rates we achieved were higher than the previous published reports.^23^, ^28^

In Elekta linacs during VMAT delivery the TCS software item checks that the dose rate is not less than 75% of the calculated value. The beam will terminate if the dose rates exceeds this limit showing an interlock “LOW DOSE MON”. We encountered no interlocks at or above 20 MU/min dose rate during the VMAT delivery. The beam characteristics reported in this study primarily focused on 6 MV X‐rays because 6 MV is the energy commissioned for IMRT and VMAT in our clinic, but we did examine other energies in preliminary tests. The output, linearity, flatness, and symmetry at low dose rates did not show significant differences between 6 MV and higher energies (10 and 15 MV). This gives confidence that the findings and methodology we described are applicable across photon energies.

PLDR showed favorable clinical outcomes in tumor control and acute/late normal tissue toxicities, as reported by many investigators.^8^, ^14^, ^15^, ^16^, ^17^, ^18^, ^19^, ^20^, ^24^ Elekta Versa HD offers several preset dose rate values; our study utilized the 20 MU/min. We shall also use any other low dose rates in the 20–100 MU/min range depending on the effective dose rate we want to achieve in the delivery. The sample size is the limitation of this study. The validity of the results can be enhanced by increasing the sample size. However, the results we obtained for ten patients were meaningful. Achieving minimum MU for small PTVs would be challenging during the initial testing. PLDR VMAT planning and delivery will become easier if the Elekta linacs allow VMAT delivery with the maximum dose rate of 10–15 MU/min. Further investigations are required to utilize the low dose rate VMAT delivery for the reirradiation of tumors in the thorax, abdomen, or pelvic region and VMAT‐based total body irradiation using Elekta solutions. Reirradiation with PLDR has been shown to be a promising radiotherapy technique for recurrent gliomas. Prospective studies with clinical outcome assessments are necessary to validate the impact on toxicity reduction in PLDR compared to standard treatment. It is worth noting that the dose rate varies drastically in the voxels of target and normal tissue across the dose calculation volume during VMAT delivery. If the planning system in the future include calculation of dose rate distribution in target and OARs, it may have significant impact in PLDR treatment planning.

## 5 CONCLUSION

Our study investigated the accuracy of low dose rate VMAT delivery and the feasibility of using the Monaco planning system to deliver PLDR VMAT without the need for 10 subfractions and beam intervals. For Elekta linacs, plan optimization at the target dose rate is not needed at the planning stage, but it can be set in the Mosaiq R&V system at the time of delivery. Low dose rate VMAT delivery is feasible on Elekta Versa HD linac with higher accuracy in the delivered dose. We believe that the method described in our study would be helpful for centers with Elekta systems looking forward to establishing PLDR.

## AUTHOR CONTRIBUTIONS

**Surendran Jagadeesan**: Conceptualization; methodology; software; validation; formal analysis; investigation; writing—original draft; writing—review & editing; visualization; data curation. **S.P. Vjaya Chamundeeswari**: Conceptualization; validation; investigation; resources; data curation; writing—review & editing; visualization

## CONFLICT OF INTEREST STATEMENT

Declaration of AI‐assisted technologies in the writing process

During the preparation of this work, the author used Grammarly in order to correct spelling, grammar, punctuation, clarity, and readability. After using this tool, the author reviewed and edited the content as needed and takes full responsibility for the content of the publication.

## Data Availability

Research data are stored in an institutional repository and will be shared upon request to the corresponding author.
